# Characteristics and Follow-Up of 13 pedigrees with Gitelman syndrome

**DOI:** 10.1007/s40618-018-0966-1

**Published:** 2018-11-10

**Authors:** F. Zhong, H. Ying, W. Jia, X. Zhou, H. Zhang, Q. Guan, J. Xu, L. Fang, J. Zhao, C. Xu

**Affiliations:** 1 Department of Endocrinology and Metabolism, Shandong Provincial Hospital Affiliated to Shandong University, 324, Jing 5 Road, Jinan, 250021 Shandong China; 2 Institute of Endocrinology, Shandong Academy of Clinical Medicine, Jinan, 250021 Shandong China; 3 Shandong Clinical Medical Center of Endocrinology and Metabolism, Jinan, 250021 Shandong China

**Keywords:** Gitelman syndrome, Pedigree, Solute carrier family 12, member 3 (SLC12A3), Genotype, Phenotype, Follow-up

## Abstract

**Context:**

Gitelman syndrome (GS) is clinically heterogeneous. The genotype and phenotype correlation has not been well established. Though the long-term prognosis is considered to be favorable, hypokalemia is difficult to cure.

**Objective:**

To analyze the clinical and genetic characteristics and treatment of all members of 13 GS pedigrees.

**Methods:**

Thirteen pedigrees (86 members, 17 GS patients) were enrolled. Symptoms and management, laboratory findings, and genotype–phenotype associations among all the members were analyzed.

**Results:**

The average ages at onset and diagnosis were 27.6 ± 10.2 years and 37.9 ± 11.6 years, respectively. Males were an average of 10 years younger and exhibited more profound hypokalemia than females. Eighteen mutations were detected. Two novel mutations (p.W939X, p.G212S) were predicted to be pathogenic by bioinformatic analysis. GS patients exhibited the lowest blood pressure, serum K^+^, Mg^2+^, and 24-h urinary Ca^2+^ levels. Although blood pressure, serum K^+^ and Mg^2+^ levels were normal in heterozygous carriers, 24-h urinary Na^+^ excretion was significantly increased. During follow-up, only 41.2% of patients reached a normal serum K^+^ level. Over 80% of patients achieved a normal Mg^2+^ level. Patients were taking 2–3 medications at higher doses than usual prescription to stabilize their K^+^ levels. Six patients were taking spironolactone simultaneously, but no significant elevation in the serum K^+^ level was observed.

**Conclusion:**

The phenotypic variability of GS and therapeutic strategies deserve further research to improve GS diagnosis and prognosis. Even heterozygous carriers exhibited increased 24-h Na^+^ urine excretion, which may make them more susceptible to diuretic-induced hypokalemia.

**Electronic supplementary material:**

The online version of this article (10.1007/s40618-018-0966-1) contains supplementary material, which is available to authorized users.

## Introduction

Gitelman syndrome (GS, OMIM 263800), one of the most common hereditary disorders of potassium homeostasis, is characterized by hypokalemia, hypomagnesemia, and hypocalciuria without hypertension [1, 2]. The prevalence of GS is approximately 1–10 per 40,000 people, and, accordingly, the prevalence of heterozygotes is approximately 1% in Western countries [3, 4]. In Asia, the prevalence of GS increases to an astonishing 10.3 per 10,000 people [5], and the prevalence of mutations may be as high as 3% [3].

Inherited in an autosomal recessive pattern, GS is caused by inactivating mutations in the solute carrier family 12 member 3 gene (SLC12A3, Gene ID: 6559; MIM: 600968; Gene Bank: NC_000016.10), which encodes the thiazide-sensitive sodium-chloride cotransporter (NCC) [6]. To date, 453 mutations have been deposited in the Human Gene Mutation Database (HGMD, http://www.hgmd.cf.ac.uk/). Interestingly, the phenotype of GS patients is highly heterogeneous [7]. Some researchers have analyzed the clinical and genetic characteristics in unrelated patients with GS. However, a comprehensive genotype and phenotype correlation has not been well established. Moreover, pedigree members have identical geographic backgrounds and similar dietary habits. The careful study of GS pedigrees may help to obtain more valuable information about this disorder.

The long-term prognosis of GS is considered to be favorable. However, hypokalemia and hypomagnesemia in GS are difficult to cure. Patients exhibit a significantly reduced quality of life due to the presence of several unspecific symptoms, such as salt cravings, thirst, dizziness, fatigue, muscle weakness, cramps, paresthesias, nocturia, polydipsia, and polyuria. Compared with the typical population, patients with GS may be at increased risk for the development of chronic kidney disease and type 2 Diabetes [8]. Therefore, follow-up study is imperative for in-depth research to seek out a better method for GS treatment and to prevent further exacerbation of the disease.

Thus, we analyzed the clinical and genetic characteristics among all the members of 13 GS pedigrees, and continuous follow-up was performed for about 3 years to accumulate valuable data on the treatment of GS. Our research may assist in the understanding of phenotypic variability and provide useful insight into the characteristics of GS as well as therapeutic strategies for this disease.

## Subjects and methods

### Ethics statement

This study was approved by the ethics committee of Shandong Provincial Hospital affiliated to Shandong University, and all the participants have signed the written informed consent before participation. The study was performed in accordance with the Helsinki Declaration.

### Subjects

**Fig. 1:**
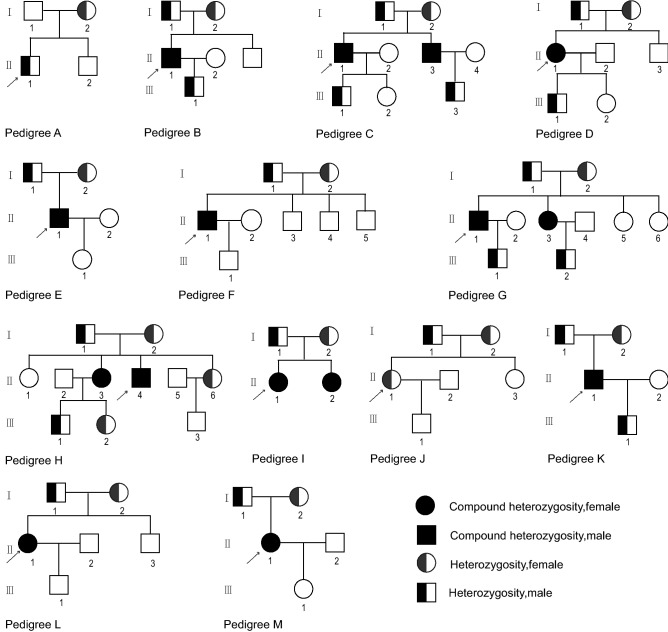
Chinese GS Pedigrees studied. Males and females are indicated by squares and circles. Affected individual is indicated by filled symbols. The proband is indicated by arrows

The study group consisted of 13 pedigrees, including 86 members, of whom 17 were GS patients (9 males and 8 females, age 39.5 ± 11.9 years) (Fig. 1). Diagnostic criteria for GS include the following: hypokalemia (serum K^+^ < 3.5 mmol/L) with inappropriate renal potassium wasting; hypomagnesemia (serum Mg^2+^ < 0.7 mmol/L) with inappropriate renal magnesium wasting; hypocalciuria (urine Ca^2+^/Cr ratio < 0.2 mmol/mmol); metabolic alkalosis; high plasma renin activity or levels; low or normal–low blood pressure [1]. None of them had history of long-term use of diuretics and laxatives or alcohol or drug addiction. Other extrarenal and renal causes of hypokalemia such as Cushing’s syndrome, primary hyperaldosteronism, tubular acidosis, history of nephrotoxic drugs or liquorice intake, stenosis of the renal artery and transcellular shift of K^+^ such as thyrotoxic or familial periodic paralysis, the use of bronchodilators, were also excluded.

### Clinical data analysis

We collected the clinical symptoms of all patients and laboratory findings among all the members of 13 GS pedigrees. Clinical symptoms include general (fatigue, dizziness/vertigo, fainting, and exercise intolerance), musculoskeletal (weakness, muscle stiffness or pain, muscle cramps, carpopedal spasm/tetany, and paralysis), renal (nocturia, polyuria, polydipsia, thirst, enuresis, and salt craving), gastrointestinal (vomiting, constipation, and abdominal pain), cardiovascular (palpitation and chest pain), and neurological symptoms (paresthesia and tremor) as previously described [9]. Severe GS was defined if patients showed at least three category symptoms at the same time, while mild GS was limited to fewer than three category manifestations. Biochemical values included concentration of serum K^+^, Mg^2+^, urine Ca^2+^/Cr, 24-h urinary Ca^2+^, Na^+^, K^+^ levels and blood pressure.

### Mutation analysis of the SLC12A3 gene

Genomic DNA was isolated from peripheral blood using genomic DNA kit (TIANGEN BIOTECH, DP 304-03). Twenty-two pairs of primers were generated to amplify the whole SLC12A3 gene, including all exons and intron–exon boundaries. Polymerase chain reaction (PCR) was performed in a 50 μL system including 4 μL dNTP, 5 μL 10 × PCR buffer, 0.3 μL Taq Hot Start (Takara Bio, Ohtsu, Japan), 4 μL genomic DNA and 1 μl forward and reverse primers. The reaction condition contained an initial denaturation step at 94 °C for 5 min subsequently followed by 40 cycles with denaturation at 94 °C for 30 s, annealing at 60 °C for 30 s and elongation at 72 °C for 30 s. PCR products were directly sequenced on an ABI 3730XL DNA sequencer (Applied Biosystems, Inc., Foster City, Calif., USA). Sequence analysis was finished through Auto Assembler software Chromas 2.0. Recurrent mutation was defined as the same mutation reappearing in at least two unrelated pedigrees.

### Bioinformatic analysis

We performed sequence alignment of NCC homologous proteins on eight species to confirm the conservation of mutated positions. To determine potential effects of the two novel mutations on NCC function, online softwares such as Mutation Taster (http://www.mutationtaster.org/), Poly Phen-2 (http://genetics.bwh.arvard.edu/pph2) and SIFT (http://sift.jcvi.org/) were employed. Modeling of wild type and mutant protein was achieved using I-TASSER workspace (https://zhanglab.ccmb.med.umich.edu/I-TASSER/), and PyMOL Viewer was used to visualize the effect of novel mutations on the protein configuration of NCC.

### Follow-up studies

All patients were reviewed by phone every 2 months to record symptoms. Biochemical examinations were performed in local hospitals every 6 months, and face-to-face follow-ups in our hospital were completed once a year. If there were special circumstances (such as hypokalemic acute attack), patients were taken to a nearby hospital for examination and treatment. The other members of all pedigrees were asked about the symptoms every 6 months and tested above the biochemical indices once a year. Pedigrees were followed for 1.9 ± 1.1 years. We also closely tracked the occurrence of potential complications and evolution such as glucose metabolism and impaired renal function.

### Statistical analyses

Values were expressed as the mean ± SD. Student’s *t* test and Fisher’s exact test were performed to compare the variables between the male and female patients. To determine the correlation between genotypes and phenotypes, Student’s *t* test was used to compare their differences. We used one-way ANOVA to explore the effect of SLC12A3 mutations on blood pressure and laboratory parameters in all pedigree members. *P* < 0.05 was considered statistically significant.

## Result

### Characteristics of the patients

As shown in Table 1, the average ages at onset and diagnosis of GS were 27.6 ± 10.2 years and 37.9 ± 11.6 years, respectively. The duration from onset to diagnosis was 10.4 ± 9.4 years, indicating a long period of underdiagnosis. Interestingly, the age of onset in males was 10 years earlier than that in females (male vs. female, 22.7 ± 8.8 years vs. 33.9 ± 8.6 years, *P* < 0.05). Clinically, muscle paralysis (47%), fatigue (41.2%), paresthesia (35.3%), muscle weakness (29.4%), palpitation (23.5%), cramps (17.6%), and tetany (17.6%) were common symptoms. Usually, muscle paralysis was the primary presenting symptom in affected males. In contrast, fatigue was more common in females (*P* < 0.05). Six patients (35.3%) had severe symptoms, with men accounting for most of them (five males, one female), which indicated that males had more severe symptoms than females. One male (patient pedigree FII-1) and one female (patient pedigree GII-3) were virtually asymptomatic.

**Table 1:** Phenotype of patients in the GS pedigrees

Pedigree	Patient	Age at onset/diagnosis (yr)	K^+^ (mmol/L)	Mg^2+^ (mmol/L)	Urine Ca^2+^/Cr (mmol/mmol)	Urinary Ca^2+^ (mmol/24 h)	Urinary Na^+^ (mmol/24 h)	Urinary K^+^ (mmol/24 h)	BP (mmHg)	Clinical presentations
A	II 1(M)	15/16	2.60	0.70	0.24	2.83	258.4	111.72	105/66	Paralysis, muscle weakness
B	II 1(M)	21/45	2.50	0.52	0.14	1.54	401.5	97.7	110/76	Paralysis, muscle weakness, palpitation, thirst, polydipsia, polyuria
C	II 1(M)	14/30	2.10	0.44	0.04	1.23	302.7	123	113/75	Paralysis, muscle weakness, palpitation, paresthesia, fainting, vertigo
	II 2(M)	25/32	2.00	0.60	0.06	1.03	352	87	104/65	Paralysis, muscle weakness, fainting
D	II 1(F)	25/48	3.22	0.38	0.04	0.36	282	100.4	108/76	Paresthesia, tetany
E	II 1(M)	26/26	2.30	0.46	0.04	1.06	237.4	106.41	96/62	Cramps
F	II 1(M)	41/65	2.60	0.62	0.16	0.84	237	108.36	110/62	Asymptomatic
G	II 1(M)	21/30	2.31	0.64	0.12	1.10	263.5	133.95	105/62	Paralysis, muscle weakness, tetany, palpitation, QT elongation, paresthesia
	II 3(F)	△/40	3.4	0.80	0.31	2.84	387.9	129.8	106/70	Asymptomatic
H	II 3(M)	28/45	2.40	0.31	0.04	2.91	335.4	94.1	108/75	Paralysis, tetany, cramps, palpitation, paresthesia
	II 2(F)	48/50	3.00	0.40	0.10	0.5	398.3	107	107/70	Fatigue
I	II 1(F)	28/43	2.70	0.59	0.05	0.69	415.25	123	101/61	Fatigue, cramps, paresthesia
	II 2(F)	40/45	2.20	0.60	0.06	1.98	354.4	128	107/65	Fatigue, paresthesia
J	II 1(F)	39/39	2.52	0.31	0.13	1.56	240	114	105/64	Paralysis, fatigue
K	II 1(M)	13/33	2.00	0.44	0.11	1.6	372.5	107.25	106/66	Paralysis, muscle pain, fatigue, paresthesia
L	II 1(F)	31/32	2.30	0.56	0.06	0.98	230.75	87.9	110/71	Fatigue, palpitation
M	II 1(F)	26/26	2.80	0.52	0.04	0.49	423.3	123	112/75	Fatigue

The normal value of blood potassium 3.5–5.5 mmol/L. The normal value of blood magnesium 0.7–1.0 mmol/L

*M* male, *F* female, *yr* year, *–* not measured, △ asymptomatic

Hypokalemia (2.53 ± 0.40 mmol/L) was present in all the patients. Among them, approximately 50% of the patients had severe hypokalemia and 35.3% of them had moderate hypokalemia. Hypomagnesemia was also found in the majority of the patients, with an average level of 0.52 ± 0.14 mmol/L. Similarly, urine Ca^2+^/Cr decreased to less than 0.2 in 88.2% of patients, with two exceptions. Consistent with clinical symptoms, males had more significant hypokalemia than females (2.31 ± 0.24 mmol/L vs. 2.77 ± 0.43 mmol/L, *P* < 0.05), while there was no significant difference in the plasma Mg^2+^ (*P* > 0.05) and urine Ca^2+^/Cr (*P* > 0.05) levels between men and women. All patients had normal blood pressure.

### Genotype analysis

**Fig. 2:**
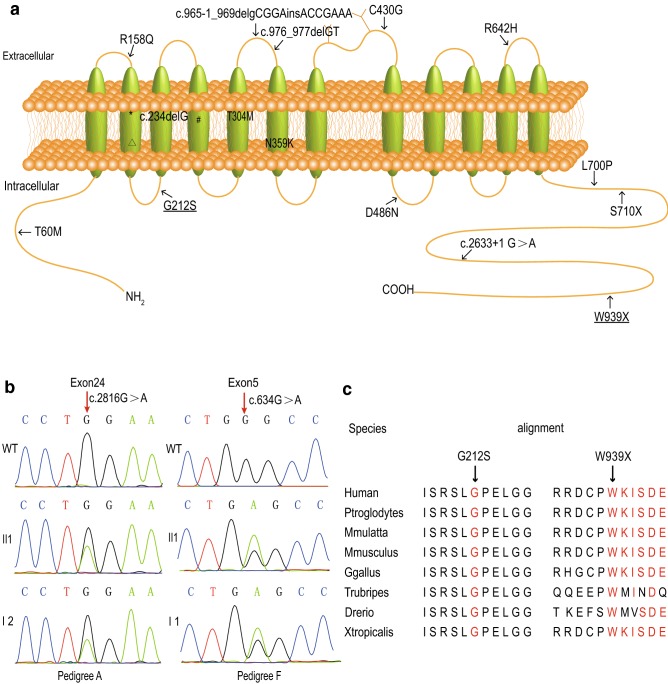
Mutation analysis of SLC12A3. **a** Schematic diagram of the NCC and mutations identified in 13 Chinese GS pedigrees. NCC is represented as a 12-transmembrane-domain protein with intracytoplasmic amino and carboxyl termini. The sites of mutations are denoted by arrows and special marks like *, Δ, #. Novel mutants are underlined. *c.486_490delinsA, ^Δ^c.506-1G >A, ^#^c.805_806insTTGGCGTGGTCTCGGTCA. **b** Two novel mutations. **c** Cross-species conservation of SLC12A3 around G212 and W939X

A total of 18 mutants were detected (Fig. 2a). All detected mutations are summarized in Table 2. There were nine missense, two nonsense, three deletion, one insertion, one insertion and deletion, and two splice-site/intronic mutations which incurred in intron–exon boundaries. In agreement with previous reports, the majority of our patients had a compound heterozygous mutation on two alleles. Nevertheless, one patient (pedigree M II-1) had three NCC mutations, and two patients (pedigree A II-1 and J II-1) from two different families had only one heterozygous mutation. We found four recurrent mutations, including T163 fs, c.506-1G > A, D486 N and T60 M, which means that these mutations were probably common in Chinese patients with GS.

**Table 2:** Genotype in 13 Chinese GS pedigrees

Pedigree	Patient	Location	Mutation
A	II 1(M)	Exon1, c.2816G > A	W939X (Hetero)
B	II 1(M)	Exon7, c.911C > T/exon 17, c.2099T > C	T304M/L700P(CH)
C	II 1(M)	Exon12, c.1456G > A/intron 22, c.2633 + 1 G > A	D486N/SP(CH)
	II 2(M)	Exon12, c.1456G > A/intron 22, c.2633 + 1 G > A	D48 N/SP(CH)
D	II 1(F)	Exon3, c.486_490delinsA/exon12, c.1456G > A	T163fs/D486N(CH)
E	II 1(M)	Exon6,c.805_806insTTGGCGTGGTCTCGGTCA/exon10,c.1288T > G	T269fs/C430G(CH)
F	II 1(M)	Exon3, c.473G > A/exon5, c.634G > A	R158Q/G212S(CH)
G	II 1(M)	Exon1, c.234delG/exon1,c.179C > T	E7 fs/T60M(CH)
	II 3(F)	Exon1, c.234delG/exon1,c.179C > T	E78fs/T60M(CH)
H	II 3(M)	Exon3, c.486_490delinsA/exon15, c.1925G > A	T163fs/R642H(CH)
	II 2(F)	Exon3, c.486_490delinsA/exon15, c.1925G > A	T163fs/R642H(CH)
I	II 1(F)	Exon1, c.179C > T/intron3, c.506-1G > A	T60M/SP(CH)
	II 2(F)	Exon1, c.179C > T/intron3, c.506-1G > A	T60M/SP(CH)
J	II 1(F)	Exon3, c.486_490delinsA	T163fs (Hetero)
K	II 1(M)	Intron3, c.506-1G > A/exon17, c.2129C > T	SP/S710X(CH)
L	II 1(F)	Intron3, c.506-1G > A/exon8, c.1077C > G	SP/N359K(CH)
M	II 1(F)	Exon3, c.486_490delinsA/exon8, c.965-1 _969delinsACCGAAA, c.976_977delGT	T163fs/fs/V326fs(CH, triple)

*M* male, *F* female, *Homo* homozygosity, *Hetero* heterozygosity, *CH* compound heterozygosity, *SP* splicing mutation

**Fig. 3:**
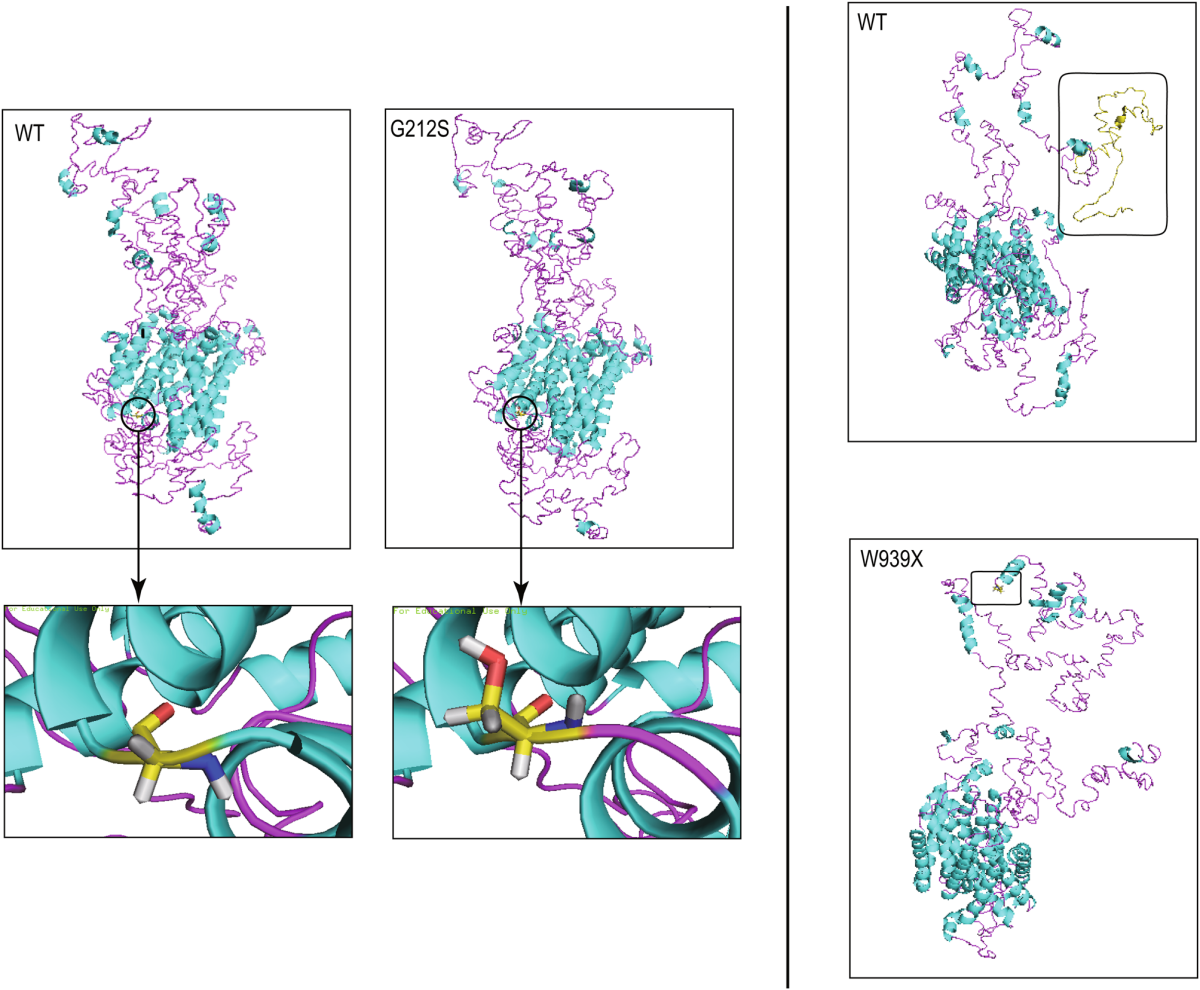
Protein structure prediction of wild-type and mutant SLC12A3

Interestingly, two novel mutations were identified in pedigree A patient II-1 (c.2816G > A, p.W939X) and pedigree F patient II-1 (c.634G > A, p.G212S). As shown in Fig. 2b, patient A II-1 inherited the only variant from his mother, while in patient F II-1, the novel variant was from his father and the other one from his mother. The missense mutation, G > A at nucleotide 634, led to a predicted amino acid substitution from glycine to serine at codon 212 of the proband in a heterozygote state. This mutation was strongly predicted to be pathogenic using three web-based programs—Mutation Taster, PolyPhen-2, and SIFT. The nonsense mutation (c.2816G >A) was predicted to result in a premature stop codon at amino acid 939 (p.W939X), leading to a truncated protein, which was 83 amino acids shorter than the wild type. This mutation was predicted to be probably damaging by Mutation Taster. According to the sequencing alignment, the locations of both G212 and W939 were highly conserved among all eight species (Fig. 2c). Moreover, both p.G212S and p.W939X were predicted to alter the protein’s three-dimensional structure by the I-TASSER workspace program (Fig. 3). G212S led to the exposure of carbon bonds between two alpha helices, which may affect the modification of NCC protein or its interaction with other proteins. W939X caused the deletion of 83 amino acids at the carboxyl terminus, so its three-dimensional structure changed significantly, especially the carbon terminal conformation. All of this information suggested that these two novel mutations can destroy the structure of NNC, leading to functional defects.

### Correlation between phenotype and genotype among patients

Based on the type of genotype, we further stratified patients into subgroups. As shown in Table 3, serum potassium levels were lower in patients with intronic mutations (intronic mutations vs. nonintronic mutations, 2.22 ± 0.26 vs. 2.70 ± 0.37 mmol/L, *P* < 0.05) or nonframeshift mutations (frameshift mutations vs. nonframeshift mutations, 2.74 ± 0.43 vs. 2.33 ± 0.27 mmol/L, *P* < 0.05). However, there was no statistical significance in the levels of 24-h urine potassium between these two groups of mutations. Though recurrent mutations were associated with more severe clinical symptoms, there was no significant difference in laboratory findings compared to other mutations. We did not detect any significant differences in serum magnesium levels, urinary potassium excretion, and blood pressure between the subgroups.

**Table 3:** Biochemical data in different mutation types

	Mutation type		Mutation type		Mutation type	
	Intronic (*n* = 6)	Nonintronic (*n* = 11)	Frameshift (*n* = 8)	Nonframeshift (*n* = 9)	Recurrent (*n* = 13)	Nonrecurrent (*n* = 4)
K^+^ (mmol/L)	2.22 ± 0.26*	2.70 ± 0.37*	2.74 ± 0.43*	2.33 ± 0.27*	2.60 ± 0.40	2.30 ± 0.36
Mg^2+^ (mmol/L)	0.54 ± 0.08	0.51 ± 0.16	0.48 ± 0.17	0.56 ± 0.85	0.52 ± 0.15	0.53 ± 0.07
Urinary K^ +^ (mmol/24 h)	109.36 ± 18.35	111.49 ± 12.84	113.58 ± 4.22	108.21 ± 15.06	112.18 ± 16.15	106.05 ± 5.98
Sp (mmHg)	107 ± 4	107 ± 4	106 ± 5	107 ± 4	106 ± 4	108 ± 4
Dp (mmHg)	67 ± 5	69 ± 6	69 ± 6	67 ± 5	68 ± 6	69 ± 5

*Sp* systolic pressure, *Dp* diastolic pressure

*P < 0.05

### Analysis of clinical and genetic characteristics among all the pedigree members

**Fig. 4:**
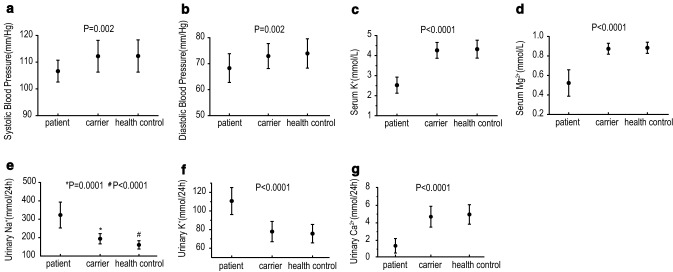
Clinical and genetic characteristics analysis among all the pedigree members. The mean ± SD values of laboratory parameters are shown for patient, carrier, and health control. *P* values for ANOVA among different genotype classes are indicated. *P value between patient and carrier, ^#^*P* value between carrier and health control

According to the genotyping and clinical symptoms, pedigree members were divided into three groups: patient (*n* = 17), carrier (*n* = 35), and healthy control (*n* = 34). Laboratory values of all the members according to different genotypic backgrounds were analyzed (Fig. 4). As expected, blood pressure and serum K^+^ and Mg^2+^ levels were significantly lower in the patient group than in the carrier and healthy control groups. The carrier group had normal blood pressure, serum K^+^ and Mg^2+^ levels, which showed no significant differences compared with the healthy control group (Fig. 4a–d). Moreover, the 24-h urinary Na^+^ level in the patient group increased significantly (*P < 0.0001, Fig. 4e). It is noteworthy that the 24-h urinary Na^+^ excretion in the carrier group was also higher than that in the healthy control group (#*P* < 0.0001, Fig. 4e). Though the 24-h urinary K^+^ level in patients obviously increased, there was no significant difference between the carrier and healthy control groups (Fig. 4f). Likewise, GS patients exhibited the lowest 24-h urinary Ca^2+^ level (*P* < 0.0001, Fig. 4g), while the carrier and healthy control groups presented similar levels of Ca^2+^ excretion. Consequently, although blood pressure, serum K^+^ and Mg^2+^ levels in heterozygous carriers were normal, 24-h urinary Na^+^ excretion increased significantly. These results suggest a potential role of sodium urine excretion in the early identification of GS.

### Follow-up

**Fig. 5:**
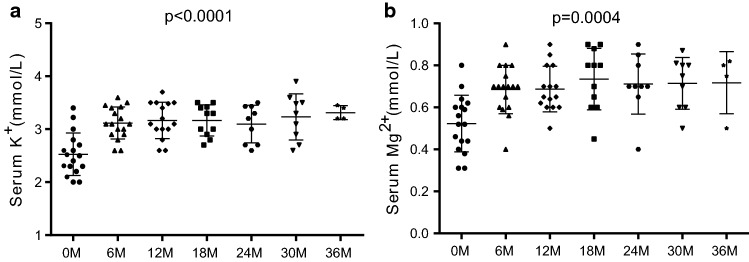
Serum potassium and magnesium levels after treatment. 0 M, 6 M, 12 M, 18 M, 24 M, 30 M, 36 M represent before treatment, 6 months, 12 months, 18 months, 24 months, 30 months, 36 months after treatment. *P* values for ANOVA among different time are indicated

During the follow-up, most of the patients took different doses of potassium supplements. As shown in Table 4 and Fig. 5, both serum potassium and magnesium levels improved significantly after treatment (*P* < 0.0001, *P* = 0.0004). However, there was no difference in serum potassium and magnesium levels at different periods of treatment.

**Table 4:** Follow-up of 13 Chinese GS pedigrees

Pedigree	Patient	Treatment				K^+^ (mmol/L)	Mg^2+^ (mmol/L)	Symptom
		Oral KCl (mmol/d)	PAMA		Sp (mg/d)			
			K (mmol/day)	Mg (mg/day)				
A	II 1(M)	40.27	8.35	106.2	–	2.90–3.00	0.70–0.90	A
B	II 1(M)	80.54	5.57	70.8	120	2.60–2.80	0.60–0.70	A
C	II 1(M)	80.54	5.57	70.8	–	3.00–3.10	0.56–0.64	A
	II 2(M)	13.42–40.27	–	–	–	3.10–3.20	0.59–0.62	A
D	II 1(F)	40.27	11.14	141.6	40	3.40–3.50	0.60–0.70	A
E	II 1(M)	40.27	8.35	106.2	–	2.90–3.30	0.70–0.80	A
F	II 1(M)	40.27	8.35	106.2	–	3.00–3.30	0.60–0.70	A
G	II 1(M)	93.10	8.35	106.2	–	3.20–3.50	0.70–0.90	A
	II 3(F)	–	–	–	–	3.40–3.50	0.80–0.90	A
H	II 3(M)	※	22.28	283.2	60	3.00–3.50	0.40–0.50	Paralysis, tetany, cramps
	II 2(F)	13.42–40.27	11.14	141.6	–	3.40–3.50	0.70–0.80	A
I	II 1(F)	53.69	5.57	70.8	80	2.70–3.00	0.60–0.70	A
	II 2(F)	53.69	5.57	70.8	80	2.60–2.90	0.60–0.70	A
J	II 1(F)	93.10	11.14	141.6	80	3.40–3.90	0.80–0.90	A
K	II 1(M)	13.42	1.86	23.6	–	2.60–2.70	0.60–0.80	A
L	II 1(F)	40.27	11.14	141.6	–	3.00–3.60	0.60–0.70	A
M	II 1(F)	60.40	5.57	70.8	–	3.00–3.10	0.70–0.80	A

Each potassium aspartate and magnesium aspartate tablet contains magnesium aspartate 0.140 g (equivalent to 11.8 mg magnesium ion) and potassium aspartate 0.158 g (equivalent to 36.2 mg potassium ion)

*M* male, *F* female, *PAMA* potassium aspartate and magnesium aspartate tablets, *Sp* spironolactone, *A* asymptomatic

^–^No supplement, ^※^Intravenous supplement of KCl 13.42 mmol/4-5 day and MgSO_4_ 2.5 g/4–5 day

Serum potassium levels increased from approximately 2.5 mmol/L to above 3 mmol/L. Serum magnesium levels almost returned to the normal range from the original level of 0.5 mmol/L. Though the majority of patients were asymptomatic, only 41.2% reached normal serum potassium levels. Compared to potassium, serum magnesium levels were easier to improve. More than 80% of patients achieved normal magnesium levels after treatment. Interestingly, patients with higher blood potassium levels mainly had higher magnesium intake. It is noteworthy that patients were taking 2–3 medicines with higher doses than usual prescription to keep their potassium steady. Six patients were taking spironolactone simultaneously, but no significant elevation in the serum potassium level was observed. Therefore, hypokalemia in GS patients was difficult to cure, while hypomagnesemia seemed easier to ameliorate.

One patient (pedigree H II-3) still suffered from periodic paralysis, tetany and cramps every 4–5 days, even while taking oral potassium magnesium aspartate (24 tablets/day) and spironolactone (3 tablets/day). He had to receive intravenous supplementation of KCl and MgSO_4_. One patient (pedigree BII-1) developed impaired fasting glucose (IFG) with fasting blood glucose 6.56 mmol/L, which was regarded as “prediabetes” [10]. Proteinuria occurred in two patients (pedigree CII-1 and II-2) with normal glomerular filtration rates and creatinine levels.

## Discussion

In this study, we summarized and analyzed the genotype and phenotype associations of all the members from 13 GS pedigrees. As expected, male patients had more severe hypokalemia and associated neuromuscular symptoms than females. Importantly, we found that the 24-h sodium urine excretion was significantly higher in heterozygous individuals than in healthy controls, though there was no difference in blood pressure levels between them. Continuous 3-year follow-up showed the difficulty of correcting hypokalemia and hypomagnesemia in GS patients, especially the former. Remarkably, we identified two novel SLC12A3 mutations that enriched the mutation database. Our findings may facilitate the understanding of the clinical and genetic characteristics of GS as well as therapeutic strategies for this disease.

Different from previous studies which focused on the genotype and phenotype of GS patients, we performed an innovatively contrastive analysis of the clinical and genetic characteristics of patients, carriers, and healthy controls in GS pedigrees. As expected, GS patients exhibited the lowest blood pressure, serum K^+^ and Mg^2+^ levels, and 24-h urinary Ca^2+^ levels compared with the carriers and healthy controls. It is worth noting that 24-h sodium urine excretion was significantly higher in the carriers than in the healthy controls, though the blood pressure was not different between them. Diet, such as the intake of Na^+^, K^+^, Ca^2+^, and Mg^2+^ may play an important role in the serum and urine levels of Na^+^, K^+^, Ca^2+^, and Mg^2+^, respectively. Salt wasting caused by diminished NCC activity leads to lower blood pressure in GS patients. Although carriers have higher 24-h sodium urine excretion than healthy controls, they do not have lower blood pressure because of self-selected higher Na^+^ intake. However, carrier children had lower blood pressures than those of the wild-type relatives because young individuals may have a lower likelihood of self-selected salt intake [11]. This suggested that heterozygous individuals may have a potential mild salt-wasting defect and we raised the possibility that the heterozygous state might underlie additional phenotypes.

As we know, the presence of hypocalciuria and hypomagnesemia is highly predictive of the clinical diagnosis of GS, but hypocalciuria and hypomagnesemia are not always present [12–15]. In our study, typical hypocalciuria and hypomagnesemia were not found in one and two patients, respectively. The reasons for normocalciuria and normomagnesemia in patients with GS remain obscure. Perhaps chronic severe hypokalemia results in secondary medullary damage and thereby compromises the function of the loop of Henle (LOH), an important site of calcium reabsorption, and induces a high distal delivery of calcium that exceeds the capacity of the DCT and connecting tubule to reabsorb this calcium [14, 16]. Like calcium, almost all filtered Mg^2+^ is reabsorbed in the LOH. Contracted extracellular fluid might lead to higher initial aldosterone levels in plasma, which could upregulate NCC in the DCT; the Mg^2+^ reabsorption that depends on the reabsorption of Na^+^ was also enhanced in DCT [14]. Moreover, some unknown Mg^2+^ regulators, such as ionic channels belonging to the transient receptor potential channel family, may diminish renal Mg2^+^ wasting caused by SLC12A3 mutation [17]. This could explain the normal urine calcium and blood magnesium observed in some patients with GS.

Previous research has shown that patients with deep intronic mutations and two mutated alleles probably had the most severe phenotype [8, 18]. In the correlation between phenotype and genotype analysis in this study, we found that patients carrying intronic or nonframeshift mutations had more severe hypokalemia. The reasons for these interesting discoveries remain incompletely understood. Maybe intronic mutations alter the splicing pattern of RNA precursors and result in the deletion of one or more exons in the mature RNA, which induces a greater loss of NCC activity. It seems difficult to explain the correlation between frameshift or nonframeshift mutation and phenotype we observed. Theoretically, the frameshift mutation should have a more severe phenotype and hypokalemia, which is contrary to the phenomenon we observed. Therefore, more patients are needed to further verify this phenomenon. In addition to the genotype’s effect on phenotype, gender differences could also account for phenotype variability. We confirmed that male patients had more severe phenotypes than females since sex hormones can control the density of NCC in the DCT cells, which leads to changes the renal excretion of electrolytes [19]. In addition, estrogens, progesterone, and PRL can increase NCC activity by increasing renal NaCl cotransporter phosphorylation [20].

It is worth noting that 58.8% of patients never reached normal potassium levels, but only 17.6% of patients never reached normal magnesium levels after treatment. Therefore, we hypothesize that hypokalemia is more difficult to correct than hypomagnesemia, which calls for in-depth research in a large population of GS. We noticed difficulty in reaching normal serum potassium and magnesium levels because a large dose of potassium can cause serious side effect, including gastric ulcers, diarrhea, and vomiting with worsening biochemistries. Furthermore, poor adherence and irregular medication may lead to a poor curative effect, which suggests that long-acting preparations, even weekly preparations, may work better. Interestingly, we found that patients with higher blood potassium levels mainly had higher magnesium intake. The insufficient supplementation of magnesium aggravates hypokalemia and renders it refractory to cure by potassium [21]. Therefore, for some patients with a poor effect of potassium supplementation, we can try to increase the dosage of magnesium supplementation. Previous studies reported that spironolactone might be helpful for hypokalemia to some degree, and spironolactone combined with potassium supplements tended to be more effective [22, 23], but we did not find an advantage of spironolactone treatment. During follow-up, one GS patient developed IFG and two patients presented proteinuria. Some studies have observed the correlation between GS and DM, suggesting that long-term hypokalemia and hypomagnesemia may lead to impaired glucose metabolism. Chronic hypokalemia results in decreased insulin secretion by holding back the closure of ATP-sensitive K + channels and L-type Ca2 + channels on the β cell surface [24, 25]. Moreover, hypomagnesemia can impair the insulin signal transduction pathway and consequently reduce the sensitivity of insulin to glucose, followed by insulin resistance [26]. In addition, the secondary hyperreninemia and hyperaldosteronism observed in GS probably cause insulin resistance [27]. However, the incidence of DM is also increasing. Therefore, the relationship between GS and DM still deserves further study. We will enlarge the sample size and extend the follow-up term to further clarify the correlation between them. GS patients are at high risk for CKD; however, the mechanism is indeed complicated and yet not well clarified. Some studies showed that chronic hypokalemia resulted in renal damage through the generation of renal tubule vacuolization, cyst formation, and tubulointerstitial nephritis [8]. However, other studies demonstrated that increasing of circulating renin, angiotensin II, and aldosterone by hypokalemia-independent volume depletion might be a more important factor of renal impairment and fibrosis [28]. Therefore, blood glucose and renal function indicators should be closely followed up in GS patients.

Notably, we identified two novel mutations (p.G212S, p.W939X) that were predicted to be pathogenic by bioinformatic analysis. Amino acid alignment analysis revealed that the glycine at position 212 and the tryptophan at position 939 were highly conserved among species. The visible differences in the whole protein configuration caused by a single base substitution further confirmed the pathogenicity of the novel mutations. All these findings indicated that the two novel SLC12A3 mutations were probably harmful and pathogenic in the GS patients. We found four recurrent mutants in SLC12A3, which would provide significant information for the screening and genetic counseling of GS. Consistent with our study, Shao, Tseng and Liu et al. also considered the missense mutations T60M and D486 N as highly frequent mutations in the Chinese population [8, 23, 29]. Although GS is inherited in an autosomal recessive manner, up to 30% of patients have simple heterozygous mutations [30]. We identified only one mutant allele in two patients. There are several possible reasons why we did not discover any mutations in the second allele: (a) existing sequencing methods can only screen exons and their intron–exon boundaries, which are incapable of finding mutations located in gene-regulating sequences such as the 5′-untranslated region and 3′-untranslated region, promoter and enhancer segments, or some harboring deep intronic mutations [16]; (b) it is difficult to identify gene sequence rearrangements involving one or more exons based on single exon analysis [31]; (c) the normal NCC protein is inactivated in some way by mutant proteins, such as kinases 1 and 4 with no lysine [32, 33]; and (d) epigenetic modifications and/or silent polymorphisms could influence the expression of the NCC [34]. In addition, one (approximately 5.8%) patient was determined to carry three SLC12A3 mutations, which was in line with other published reports [23].

There are certain limitations to our study, such as the relatively small number of pedigrees and slightly short follow-up time. Nonetheless, we are still expanding the scale of the pedigrees and extending the follow-up time to perfect our research. Moreover, conclusion on the treatment is mostly based on observational study which may seem slightly unreliable relative to random control trial (RCT) study. However, our research is rigorously designed and followed up regularly with a dedicated person responsible for regular guidance and testing. If possible, we will design a RCT study to confirm the relevant viewpoints in the future.

In conclusion, the phenotypic variability and characteristics of GS as well as therapeutic strategies merit further research to improve the diagnosis and prognosis of this disease. Moreover, our findings suggested that 24-h sodium urine excretion may be a predictor of early NCC dysfunction and SLC12A3 heterozygous individuals may be more susceptible to diuretic-induced hypokalemia.

## Electronic supplementary material

Below is the link to the electronic supplementary material.

Supplementary material 1 (DOCX 12 kb)

Supplementary material 2 (DOCX 13 kb)
