# Supplementary material for: Characteristics and Follow-Up of 13 pedigrees with Gitelman syndrome
Source: J Endocrinol Invest. 2018 Nov 10;42(6):653–65. doi: 10.1007/s40618-018-0966-1 (PMC6531408; doi:10.1007/s40618-018-0966-1)
Supplement: Supplementary file 1 — Supplementary material 1 (DOCX 12 kb) [file 40618_2018_966_MOESM1_ESM.docx]

**Supplementary Material 1. Characteristics of carriers and healthy controls**

|  | Carriers | healthy controls |
| --- | --- | --- |
| Age(yr) | 48.9 ± 24.8 | 31.7 ± 17.3 |
| Sex | 15 females, 20 males | 15 females, 19 males |
| Weight (kg) | 63.9 ± 20.3 | 60.4 ± 22.8 |
| Height (cm) | 158.3 ± 33.5 | 159.0 ± 29.2 |
