# Supplementary material for: Characteristics and Follow-Up of 13 pedigrees with Gitelman syndrome
Source: J Endocrinol Invest. 2018 Nov 10;42(6):653–65. doi: 10.1007/s40618-018-0966-1 (PMC6531408; doi:10.1007/s40618-018-0966-1)
Supplement: Supplementary file 2 — Supplementary material 2 (DOCX 13 kb) [file 40618_2018_966_MOESM2_ESM.docx]

**Supplementary Material 2. Levels of plasma renin activity and of plasma aldosterone**

| Pedigree | Patient | Renin (pg/ml) | AII(pg/ml) | ALD(pg/ml) |
| --- | --- | --- | --- | --- |
| A | II1(M) | 32.72↑ | 160.36↑ | 123.65 |
| B | II1(M) | 91.66↑ | 171.41↑ | 129.53 |
| C | II1(M) | 45.82↑ | 150.67↑ | 205.87↑ |
|  | II2(M) | 56.89↑ | 134.7↑ | 116.28 |
| D | II1(F) | 16.81↑ | 115.49 | 158.49 |
| E | II1(M) | 65.31↑ | 79.38 | 127.04 |
| F | II1(M) | 45.89↑ | 160.76↑ | 217.64↑ |
| G | II1(M) | 35.76↑ | 38.12 | 340.86↑ |
|  | II3(F) | 58.65↑ | 189.65↑ | 180.78↑ |
| H | II3(M) | 20.48 | 26.6 | 187.57↑ |
|  | II2(F) | 70.67↑ | 119.72 | 260.82↑ |
| I | II1(F) | 78.16↑ | 129.89↑ | 290.54↑ |
|  | II2(F) | 89.85↑ | 149.87↑ | 127.53 |
| J | II1(F) | 325.14↑ | 103.75 | 549.74↑ |
| K | II1(M) | 109.72↑ | 126.54 | 111.46 |
| L | II1(F) | 77.64↑ | 139.47↑ | 199.73↑ |
| M | II1(F) | 89.07↑ | 141.61↑ | 110.71 |
| Average |  | 77.07 ± 69.15↑ | 125.76 ± 43.74 | 202.25 ± 112.01↑ |

Abbreviations: AII = angiotensin II; ALD = aldosterone.

The normal value of renin **4-24** pg/ml (clinostatism). The normal value of AII **25-129** pg/ml (clinostatism). The normal value of ALD 10-160 pg/ml (clinostatism). All the test indexes were obtained in the decubitus position.

It was obvious that renin angiotensin aldosterone system (RAAS) was activated in GS patients.
